# Association of Hypertension With Both Occurrence and Outcome of Symptomatic Patients With Mild Intracranial Atherosclerotic Stenosis: A Prospective Higher Resolution Magnetic Resonance Imaging Study

**DOI:** 10.1002/jmri.27516

**Published:** 2021-03-10

**Authors:** Zhang Shi, Ming Zhao, Jing Li, Zakaria Meddings, Yibing Shi, Tao Jiang, Qi Liu, Benqiang Deng, Jianping Lu, Zhongzhao Teng

**Affiliations:** ^1^ Department of Radiology Changhai Hospital, Naval Medical University Shanghai China; ^2^ Department of Radiology University of Cambridge Cambridge UK; ^3^ Department of Neurology Changhai Hospital, Naval Medical University Shanghai China; ^4^ The 983th Hospital of Joint Logistics Support Forces of Chinese PLA Tianjin China; ^5^ Department of Radiology Xuzhou Central Hospital Xuzhou China; ^6^ Beijing Advanced Innovation Center for Biomedical Engineering Beihang University Beijing China

**Keywords:** intracranial disease, plaque, ischemic stroke, atherosclerosis, hypertension, magnetic resonance imaging, outcome

## Abstract

**Background:**

Intracranial atherosclerotic plaque causing mild luminal stenosis might lead to acute ischemic events. However, the difference between culprit and nonculprit lesions is unclear, as are the factors associated with favorable treatment outcomes.

**Purpose:**

To quantify characteristics of intracranial atherosclerosis with mild luminal stenosis and to identify factors associated with lesion type (culprit or nonculprit) and with clinical outcomes.

**Study Type:**

Prospective

**Population:**

293 patients who had acute stroke with mild luminal stenosis (<50%) in the middle cerebral or basilar artery.

**Field Strength/Sequence:**

3.0 T higher resolution magnetic resonance imaging (hrMRI) of intracranial arteries and whole brain MR images.

**Assessment:**

Morphological and compositional analysis of plaques was performed. This included assessment of plaque volume, plaque burden, remodeling ratio, eccentricity, intraplaque hemorrhage, and enhancement ratio. Clinical outcomes were assessed according to the modified Rankin Scale (mRS) at day 90, with a favorable outcome being defined as a 90‐day mRS ≤2.

**Statistical Tests:**

The odds ratios (ORs) with 95% confidence intervals (CIs) were calculated by a logistic regression model.

**Results:**

Hypertension (OR 5.2; 95% CI 2.6–10.3; *P* < 0.05) and hrMRI enhancement ratio (OR 2.7; 95% CI 1.4–5.1; *P* < 0.05) were independently associated with lesion type. Patients without hypertension had significantly more (*P* < 0.05) favorable outcomes (124/144) than patients with hypertension (97/149). Most hypertensive patients without any previous blood pressure control (54/63) had a favorable outcome. However, these patients were significantly younger (*P* < 0.05) than those with adequate blood pressure control. After adjusting for all significant characteristics, hypertension duration (OR 1.19; 95% CI 1.09–1.29; *P* < 0.05), hypertension management (OR 2.49; 95% CI 1.18–5.26; *P* < 0.05), and enhancement ratio (OR 0.01; 95% CI 0.001–0.157; *P* < 0.05) were found to be independent high‐risk factors for outcome prediction.

**Data Conclusion:**

hrMRI provided incremental value over traditional risk factors in identifying higher risk intracranial atherosclerosis with mild luminal stenosis.

**Level of Evidence:**

2

**Technical Efficacy:**

Stage 2

Intracranial atherosclerotic disease (ICAD) is a major cause of ischemic stroke worldwide, especially accounting for 40% of stroke in the Asian population.^1^, ^2^, ^3^ Angiology‐defined luminal stenosis remains the primary criterion for risk assessment.^4^, ^5^, ^6^ Luminal stenosis >50% has been shown to be independently associated with acute ischemic stroke.^5^ However, studies have revealed that lesions without significant luminal stenosis can also lead to acute events^7^, ^8^ as summarized in a recent meta‐analysis study in which approximately 50% of acute/subacute ischemic events were due to this type of lesion.^8^ Autopsy studies have also reported that approximately 25% of fatal ischemic strokes are attributed to intracranial plaques with only mild to moderate stenosis.^9^, ^10^

Positive remodeling is often observed in culprit lesions with mild stenosis where plaque grows outwards without causing significant lumen narrowing.^11^ These cannot be visualized by conventional luminal angiographic techniques such as computed tomography angiography (CTA), MR angiography (MRA), and digital subtraction angiography (DSA).^8^ Numerous studies have demonstrated that detailed lesion morphological and compositional features are more relevant to patient clinical presentations and subsequent ischemic events than luminal stenosis alone.^12^, ^13^, ^14^, ^15^ Advanced higher resolution MRI (hrMRI) is capable of visualizing detailed lesion structural features, including lipid‐rich necrotic core,^16^ intraplaque hemorrhage (IPH),^11^ thin/ruptured fibrous cap,^17^ and lesion inflammation.^14^, ^18^ However, despite the serious disease burden carried by intracranial atherosclerosis with mild stenosis, most studies have focused on lesions causing severe stenosis. Typically, studies have reported results on lesions with mild stenosis using only a small patient cohort.^19^, ^20^, ^21^ The clinical prevalence and lesion characteristics of those causing mild stenosis therefore requires further investigation. In addition, few studies have been carried out to identify factors associated with clinical outcomes in patients with this type of lesion.^8^

Thus the aim of this prospective study was to quantify differences in hrMRI‐defined characteristics between culprit and nonculprit intracranial atherosclerosis causing mild luminal stenosis, and to identify an optimal method to differentiate lesion types and factors associated with clinical outcomes following standard clinical management.

## Methods

### Study Design and Population

This study was approved by the Institutional Review Board of Changhai Hospital of Naval Medical University (Registration number: CHEC2018‐092) and all patients provided written informed consent. Patients attending the neurological clinic who had acute ischemic stroke (onset within 30 days) with mild intracranial stenosis (<50%) in one of or both middle cerebral artery (MCA) or basilar artery (BA), determined by CTA or MRA, were prospectively recruited from January 2013 to December 2018. All patients received standard medical treatment during hospitalization. The inclusion criteria were as follows: 1) aged 18–90 years old; 2) undergoing hrMRI examination during hospitalization within 30 days from onset; and 3) at least one high cardiovascular risk factor, including hypertension, diabetes mellitus, hypercholesterolemia, or cigarette smoking. Patients were excluded if they had: 1) nonatherosclerotic intracranial arterial disease (such as vasculitis, Moya‐Moya disease, or intracranial artery dissection); 2) suspected cardio‐embolic stroke; 3) presence of notable stenosis of the extracranial arteries (≥50%) assessed by ultrasound; 4) presence of ascending aortic arch atheroma as identified on MRI; 5) known coagulopathy, heart or respiratory failure; 6) intracranial hemorrhage; 7) renal dysfunction (serum creatinine >133 μmol/L); 8) serious disturbance of consciousness; and 9) poor image quality assessed by three readers (one with 7 years' experience of radiological diagnosis [Z.S.], another with 14 years' experience [J.L.], and the other with more than 25 years' experience [Q.L.]). The image quality including all of hrMRI images was assessed with the following method: each slice was graded on a 4‐point scale (1 = poor; 4 = excellent) based on the overall signal‐to‐noise ratio and the contrast between the vessel wall and surrounding tissues. Images with grades ≥3 with most lumen and outer wall boundaries visible were included in this analysis; outer wall boundaries were manually segmented on T2‐weighted (T2W) images at sites of maximum plaque area.

### Higher Resolution, Multi‐Contrast Magnetic Resonance Imaging Acquisition

The hrMRI was performed in two 3.0 T whole body systems (GE Signa 3.0 T HDxt, GE Healthcare, Waukesha, USA; and Skyra Siemens Healthcare, Erlangen, Germany). The GE system was equipped with an eight‐channel phased‐array head coil, and the Siemens system was equipped with a 20‐channel phased array head and neck coil. After an initial multi‐plane localizer sequence, axial three‐dimensional (3D) time‐of‐flight (TOF) MRA was performed to identify the stenosis. The hrMRI scanning parameters for both MCA and BA were: 1) 3D TOF MRA: repetition time (TR)/echo time (TE) = 29/3.4 msec, field of view (FOV) = 24 × 21 cm^2^, slice thickness = 1.2 mm, number of excitation (NEX) = 1, matrix = 384 × 192, sequence duration = 4.78 minutes; 2) T2W fast spin echo (FSE): TR/TE = 2883/50 msec, FOV = 10 × 10 cm^2^, NEX = 3, matrix = 320 × 256, echo‐train length (ETL) = 20, slice thickness = 2 mm, in‐plane image resolution = 0.32 mm × 0.39 mm, and sequence duration = 3.85 minutes; 3) T1‐weighted (T1W) FSE: TR/TE = 567/16 msec, FOV = 10 × 10 cm^2^, NEX = 2, matrix = 320 × 256, ETL = 6, slice thickness = 2 mm, in‐plane image resolution = 0.32 mm × 0.39 mm, and sequence duration = 5 minutes; 4) contrast enhancement T1‐weighted (CE‐T1W) imaging following a dose of 0.1 mmol/kg Gd‐DTPA injected by power injector at a rate of 2 mL/sec followed by 15 mL of saline solution. Twelve slices were acquired for T1W, T2W, and CE‐T1W sequences.

### MRI Data Analysis

**FIGURE 1:**
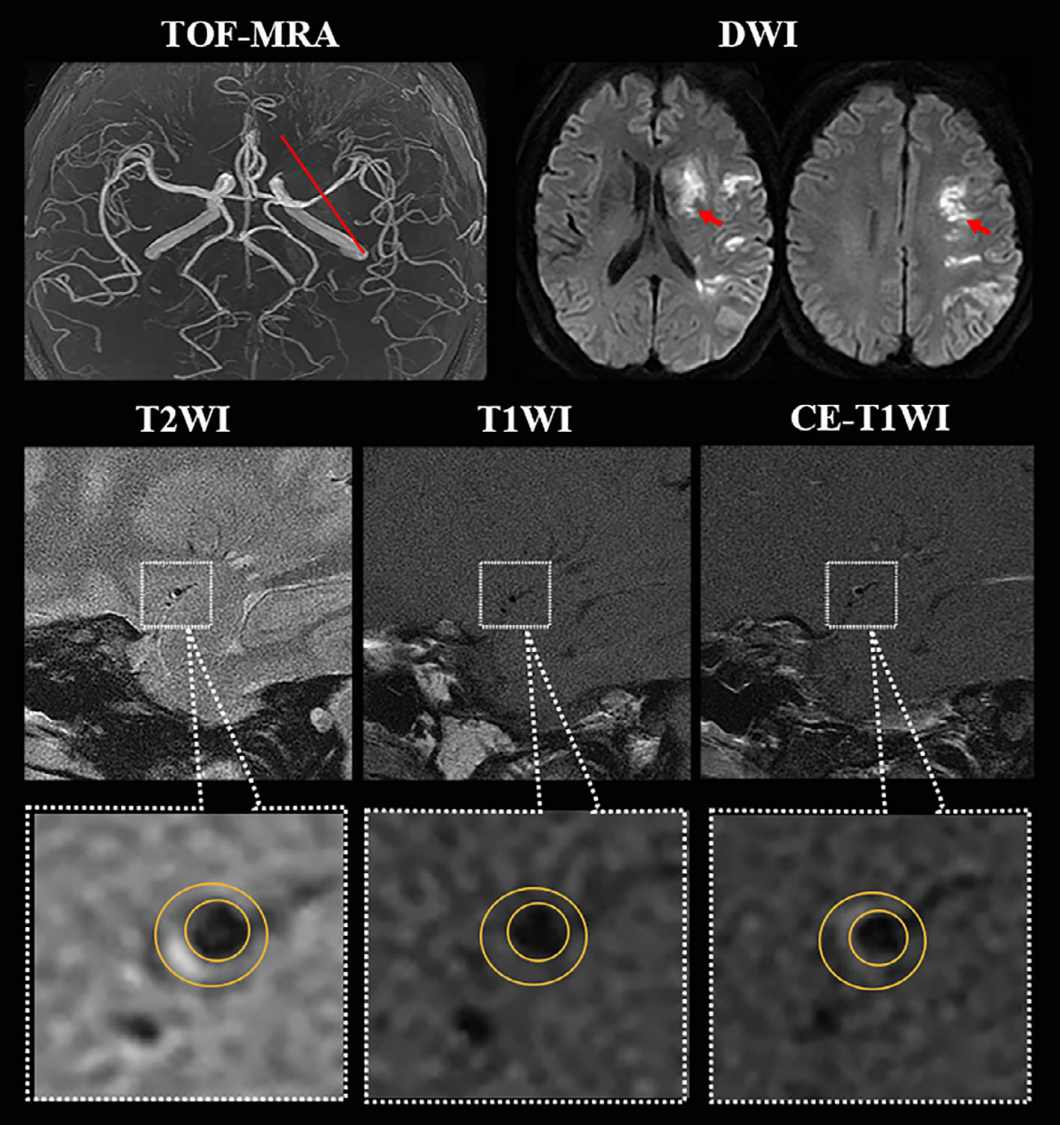
Higher resolution magnetic resonance imaging (hrMRI) images showing intracranial atherosclerotic plaque on middle cerebral artery. A 53‐year‐old man with hypertension for 10 years and without previous blood pressure management having acute ischemic stroke on the left cerebrum; TOF‐MRA demonstrated a mild stenosis (<50%) located on M1 segment of left MCA, and DWI showed a sheet acute infarcts; hrMRI images including T2WI, T1WI, and CE‐T1WI at the most stenotic site visualized the outer wall boundary (large yellow circle) and the lumen (small yellow circle), and the hrMRI parameters (minimal luminal area = 2.94 mm^2^; plaque volume = 18.98 mm^3^; plaque burden = 76.3%; remodeling ratio = 67.1%; eccentricity index = 0.51; enhancement ratio = −5.3%) were obtained; after hospitalizing management under high‐risk factor control, he had a favorable outcome with 90 mRS being 1.

**FIGURE 2:**
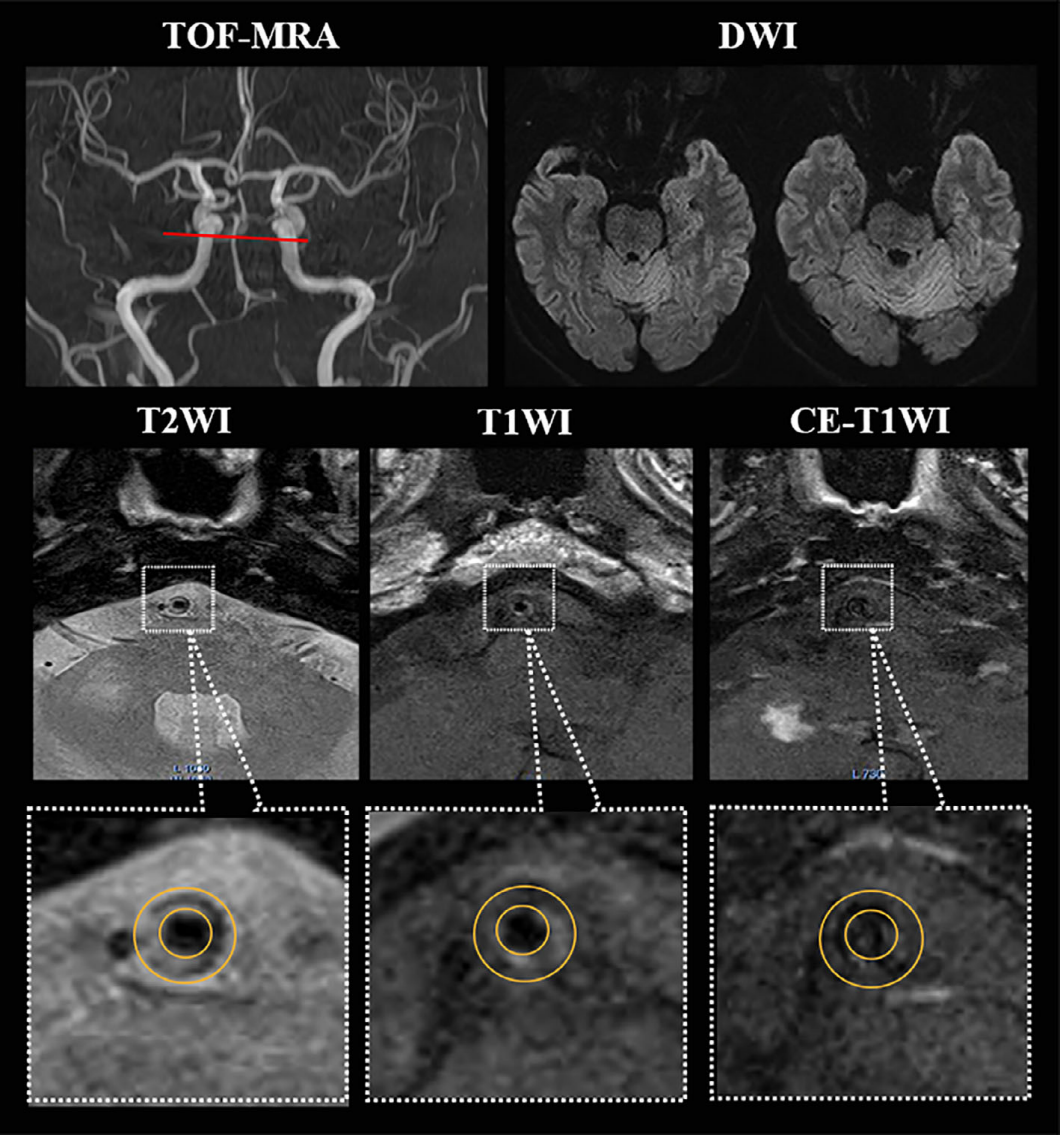
hrMRI images showing intracranial atherosclerotic plaque on basilar artery. A 56‐year‐old woman without hypertension having acute ischemic symptom with 2 weeks; TOF‐MRA showing a <50% stenosis of BA (red line on the most stenotic site) and DWI without any fresh infarct; hrMRI images showing an eccentric plaque with the nonstenotic lumen (small yellow circle); the hrMRI parameters (minimal luminal area = 2.71 mm^2^; plaque volume = 14.02 mm^3^; plaque burden = 78.8%; remodeling ratio = 71.1%; eccentricity index = 0.63; enhancement ratio = 29.5%) were obtained; after standard medical management, she had achieved 0 score of the mRS at day 90.

MR data were reviewed and analyzed by three radiologists (one with 7 years' experience [Z.S.], another with 14 years' experience [J.L.]), and the other with more than 25 years' experience [Q.L.]) who were not involved in any statistical analysis. An atherosclerotic plaque was defined as a focal wall thickening identified on hrMRI images with or without luminal stenosis. Each detected plaque was classified as culprit or nonculprit referring to patient clinical presentations and findings in diffusion weighted imaging (DWI) and fluid‐attenuated inversion recovery (FLAIR) images. The culprit plaque was identified as a lesion arising on the ipsilateral side to a fresh infarction on the DWI images with accompanying clinical symptoms. A plaque was considered to be a nonculprit plaque when it occurred in patients with presumed ischemic stroke/TIA but without an infarct on DWI and FLAIR. If more than one plaque was present in the same vascular territory, the most stenotic lesion was chosen for analysis (Figs. 1 and 2).

**FIGURE 3:**
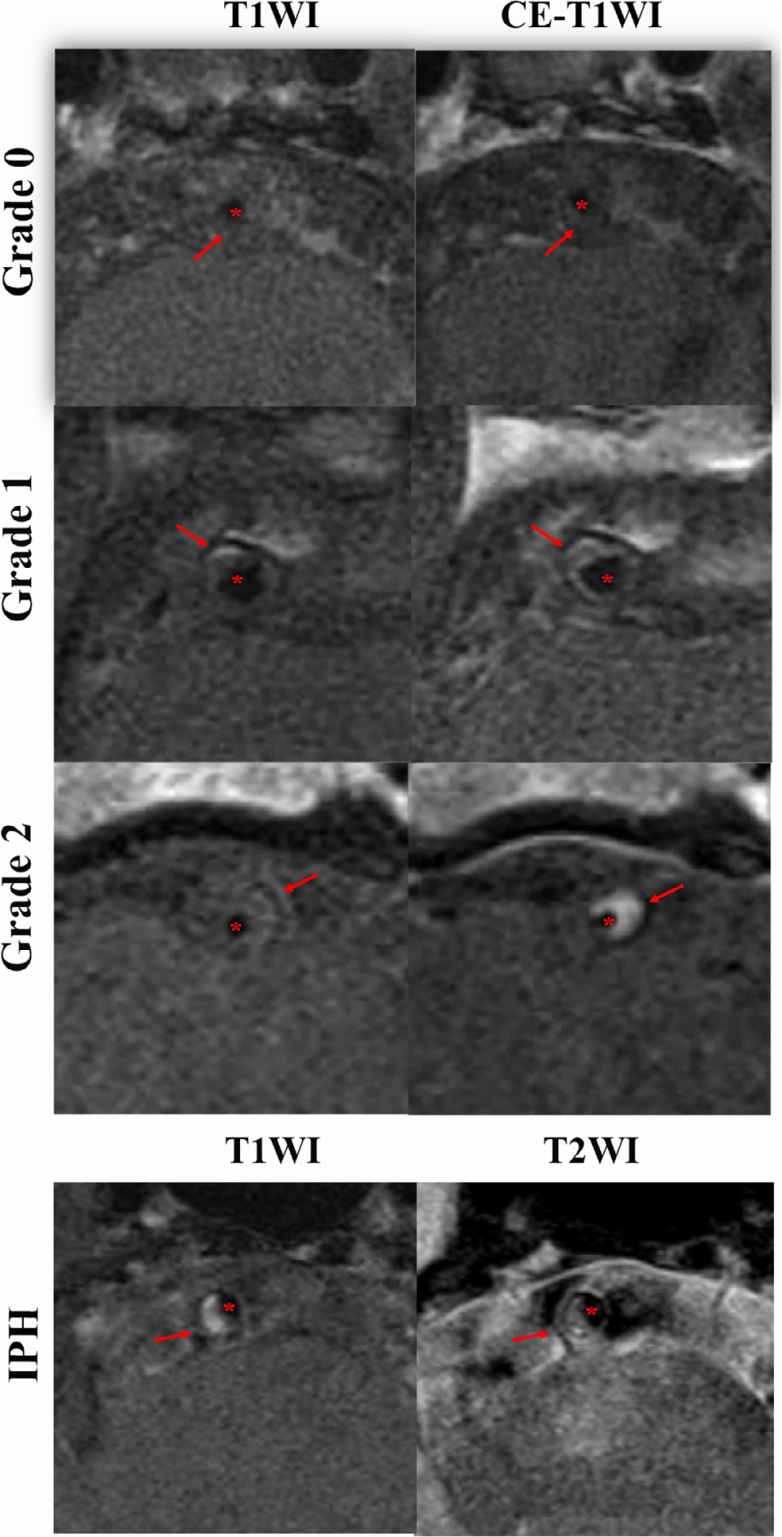
The examples of plaque enhancement grade and intraplaque hemorrhage on hrMRI. The first example is a visualization from the basilar artery using T1WI and CE‐T1WI at the most stenotic site, which includes plaque (red arrow) and the lumen (red star) with the enhancement grade gradually increasing from 0 to 2. The second case shows the hyperintensity on T1WI at the most stenotic site indicating intraplaque hemorrhage at the basilar artery, including plaque (red arrow) and the lumen (red star).

Plaque lumen and outer wall boundaries were manually segmented on T2W images using VascularView (Nanjing Jingsan Medical Science and Technology, Ltd., China). The boundary could be adjusted if necessary. The same contours were used to analyze T1W and CE‐T1W images. The degree of luminal stenosis was measured based on TOF maximum intensity projection using the WASID criterion.^22^ Minimal luminal area (MLA) was the lumen area at the most stenotic site. Plaque volume was calculated by,

$$\text{plaque volume}=\sum_{N}\left(\text{total wall area}-\text{lumen area}\right)\times \text{slice thickness}$$

where *N* is the number of slices. Plaque burden was defined as,

$$\text{Plaque burden}=\max\left({\left[1-\frac{\text{Lumen area}}{\text{Total wall area}}\right]}_{i}\times 100\%\right)$$

where *i* indicated the *i*th slice. The arterial remodeling ratio (RR) was defined,^23^

$$\mathrm{RR}=\frac{{\text{Wall area}}_{\text{lesion}}}{{\text{Wall area}}_{\text{reference}}}\times 100\%$$

and positive remodeling was defined as RR > 1.05 and negative remodeling as RR < 0.95. The eccentricity index was defined,

$$\text{Eccentricity index}=\frac{{\text{Wall}}_{\max}\;\text{thickness}-{\text{Wall}}_{\min}\;\text{thickness}}{{\text{Wall}}_{\max}\;\text{thickness}}$$

and eccentricity was assumed if the index was ≥0.5. The enhancement ratio is defined as

$$\text{Enhancement ratio}=\frac{S_{\text{postcontrast}}-S_{\text{precontrast}}}{S_{\text{pretcontrast}}}\times 100\%$$

where *S* is the signal intensity at the slice of greatest enhancement normalized by the signal from adjacent gray matter (in a region of ~15 mm^2^ at the cerebral cortex or hippocampus).^14^ The enhancement ratio was classified as: grade 0 (enhancement ratio < 15%); grade 1 (15% ≤ enhancement ratio ≤ 50%); or grade 2 (enhancement ratio > 50%). IPH was identified if the signal intensity in a region within the plaque was >150% of that in one of the adjacent muscles on precontrast T1W images (Fig. 3).

The reproducibility of the image analysis was assessed through intra‐ and interobserver studies. Sixty patients were randomly selected, and images were reviewed by one reader (**) twice with an interval of 12 weeks to avoid bias. The same MR images were independently reviewed by another two readers (** and **) for testing the inter‐observer agreement during the first time measured by the first reader.

### Clinical Data Collection

Each patient underwent standard clinical management following guidelines at admission, during the hospital stay and after discharge. Patients' demographics and disease history (age, gender, smoking, diabetes, hypertension, hyperlipidemia, etc.) and symptoms were recorded. National Institutes of Health Stroke Scale (NIHSS; range, 0 [no symptoms] to 42 [most severe neurologic deficits]) was recorded to assess the severity of neurological disorder.^24^ The modified Rankin Scale (mRS; range, 0 [normal] to 6 [death]), a parameter for disability assessment, was assessed via telephone interview at 90 days after discharge.^25^ A favorable outcome after medical treatment was defined as a 90‐day mRS of 0 to 2 indicating fully functional independence regain, while an unfavorable outcome was defined as a 90‐day mRS of 3 to 6. The history of hypertension, diabetes, hyperlipidemia, and smoking was recorded during patient interview and by reviewing the medical recorder. Patients with hypertension, diabetes, and hyperlipidemia were further grouped according to the following management types before admission: 1) no management, if the patient did not receive any treatment; 2) partial management, if the patient had irregular treatment/monitoring; and 3) strict control, when the patient received treatment and monitoring regularly for more than 6 months.

### Statistical Analysis

Statistical analyses were performed using SPSS 24.0 (IBM, USA). Normality testing was performed to assess the variable distribution. Univariable analysis (*t*‐test or Mann–Whitney *U*‐test as appropriate for the comparison of continuous variables, and chi‐squared for the comparison of categorical variables) was used to identify parameters associated with plaque type (culprit or nonculprit) and clinical outcomes (mRS ≤2 or mRS >2). Multivariable logistic regression analysis was then performed, which included variables with *P* < 0.05 in univariate tests. The odds ratios (ORs) with 95% confidence intervals (CIs) were calculated by a logistic regression model. The diagnostic performance was described using receiver operating characteristic (ROC) curves and area under curve (AUC) values. ROC curves were compared using the method developed by DeLong et al.^26^ The reproducibility of continuous variables was evaluated using the intraclass coefficient (ICC) with a two‐way random‐effects model, and the kappa value was determined for the categorical variables. *P*‐value <0.05 was considered to be statistically significant.

## Results

In total, CTA or MRA images from 446 patients with neurological symptoms were reviewed, of which 43 had dissection, 38 Moya‐Moya disease, 31 aortic arch atheroma, and 29 vasculitis. Finally, hrMRI data from 305 patients were reviewed and 12 further patients were excluded due to poor image quality. hrMRI data from 293 patients (59.1 ± 10.3 years old; 200 males) were eventually included in the study and analyzed. The patient demographics are listed in Table 1. One hundred and sixty‐nine lesions were located in the MCA with 130 being culprit, and 124 in the BA with 103 being culprit. At discharge, the mRS was 1.4 ± 1.1. Two hundred and twenty‐one (75.4%) patients had a favorable outcome after treatment (mRS ≤2) whereas 72 cases had an unfavorable outcome (mRS >2) at day 90.

**TABLE 1:** Demographics and Plaque Characteristics Between Culprit and Nonculprit Lesion

		Plaque Type, Mean ± SD or *N* (%)		
	Total, *N* = 293	Culprit, *N* = 233	Nonculprit, *N* = 60	*P*‐Value
Age (year)	59.1 ± 10.3	58.8 ± 10.9	60.2 ± 7.5	0.360
Age stage				
<45 years old	11 (9.1)	11 (10.5)	0 (0)	0.527
46–65 years old	71 (58.7)	61 (58.1)	10 (62.5)	
>65 years old	39 (32.2)	33 (31.4)	6 (37.5)	
Male	200 (68.3)	160 (68.7)	40 (66.7)	0.766
Hypertension	149 (50.9)	135 (57.9)	14 (23.3)	**<0.001**
Diabetes mellitus	83 (28.3)	63 (27.0)	20 (33.3)	0.335
Hyperlipidemia	80 (27.3)	50 (21.5)	30 (50.0)	**0.001**
Smoking	113 (38.6)	89 (38.2)	24 (40.0)	0.798
NIHSS	3.2 ± 2.5	3.3 ± 2.7	2.6 ± 1.3	**0.038**
mRS at day 90	1.8 ± 1.2	1.9 ± 1.2	1.3 ± 1.2	**0.001**
Location				
MCA	169 (57.7)	130 (55.8)	39 (65.0)	0.198
BA	124 (42.3)	103 (44.2)	21 (35.0)	
Enhancement ratio (%)	18.2 ± 20.1	16.2 ± 20.9	9.8 ± 14.2	**<0.001**
Enhancement grade				
Grade 0, <15%	109 (37.2)	84 (36.1)	25 (41.7)	**0.035**
Grade 1, 15%–50%	151 (51.5)	116 (49.8)	35 (58.3)	
Grade 2, >50%	33 (11.3)	33 (14.2)	0 (0)	
Stenosis (%)	34.8 ± 11.4	34.9 ± 11.3	34.7 ± 11.7	0.944
Intraplaque hemorrhage	44 (15.0)	43 (18.5)	1 (1.7)	**0.001**
MLA (mm^2^)	3.7 ± 3.1	3.9 ± 3.3	2.7 ± 1.8	**0.005**
Plaque volume (mm^3^)	30.8 ± 16.8	32.3 ± 18.1	24.8 ± 8.1	**0.002**
Plaque burden (%)	76.6 ± 11.3	76.3 ± 10.9	77.9 ± 12.5	0.330
Remodeling ratio (%)	110.2 ± 49.1	112.7 ± 53.6	100.7 ± 22.9	0.093
Remodeling type				
Positive remodeling	132 (53.7)	100 (50.8)	32 (65.3)	0.068
Negative remodeling	114 (46.3)	97 (49.2)	17 (34.7)	
Eccentricity	196 (66.9)	160 (68.7)	36 (60.0)	0.203

The bold numbers represented *P*‐value <0.05.

BA = basilar artery; MCA = middle cerebral artery; MLA = minimum luminal area; mRS, modified Rankin Scale; NIHSS = National Institutes of Health Stroke Scale.

In the following sections, the difference in high risk factors and lesion characteristics between culprit and nonculprit lesions was first analyzed, followed by patient cohort with different treatment outcome, and finally factors associated with unfavorable outcomes were identified.

### Patient Demographics, Plaque Characteristics, and Lesion Type

**FIGURE 4:**
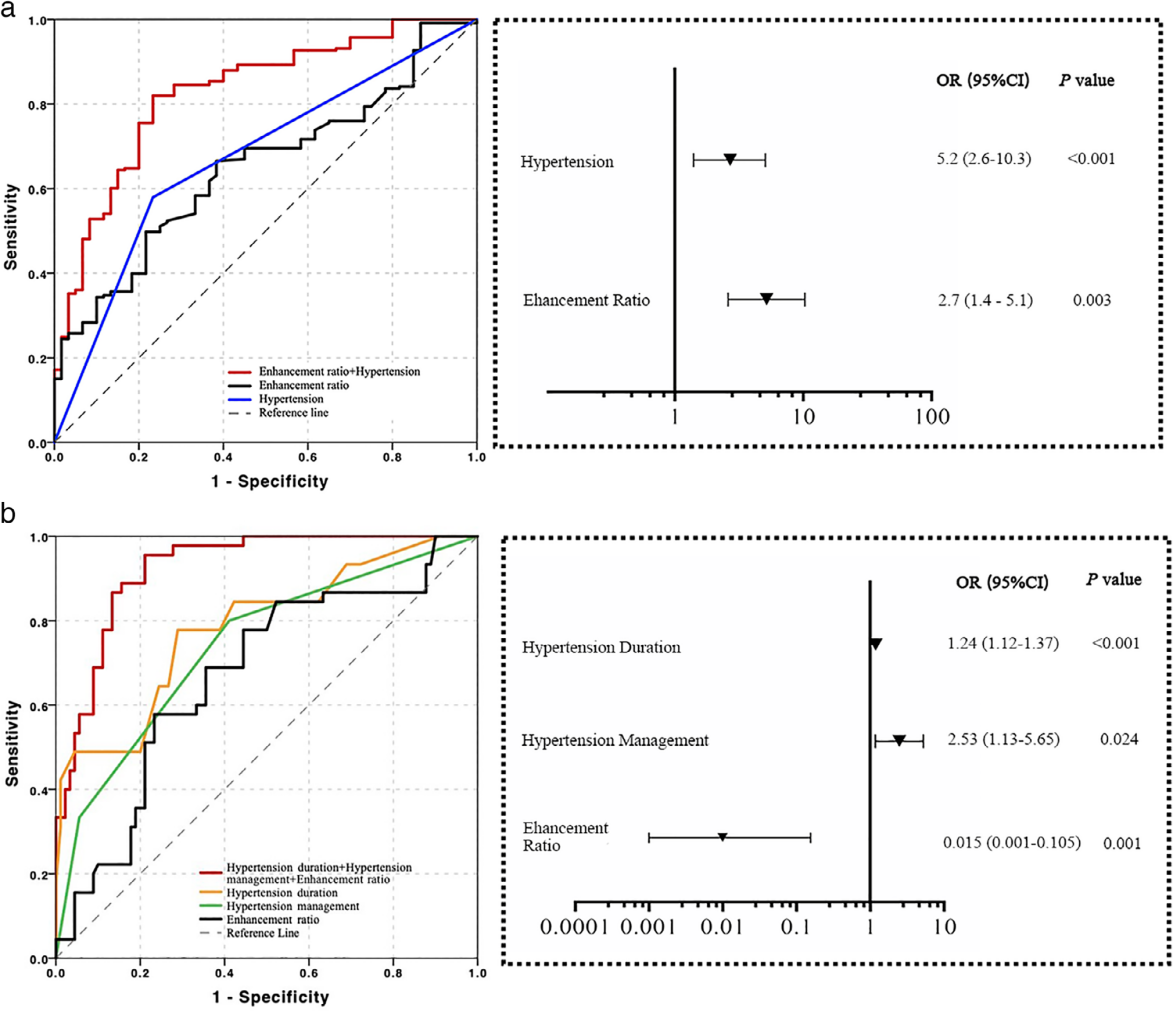
ROC curves as well as OR and 95% CI on the basis of multivariable logistic regression to (a) differentiate culprit from nonculprit lesions; (b) predict the favorable outcomes from unfavorable ones.

As summarized in Tables 1 and 3, the univariable analyses showed that hypertension, hypertension management, hyperlipidemia, NIHSS, enhancement ratio, enhancement grade, plaque volume, MLA, and presence of IPH were significantly associated with culprit lesions (all *P* < 0.05). Multivariable logistic regressions confirmed that hypertension (OR 5.2; 95% CI 2.6–10.3; *P* < 0.05) and enhancement ratio (OR 2.7; 95% CI 1.4–5.1; *P* < 0.05) were strongly associated with culprit plaques. Compared with hypertension alone (AUC =0.683 [95% CI 0.599–0.747]) or enhancement ratio alone (AUC =0.671 [95% CI 0.593–0.721]), the combination of these two factors improved the AUC to 0.829 (95% CI 0.772–0.886; *P* < 0.05) with sensitivity and specificity being 0.861 and 0.792, respectively (Fig. 4a).

### Patient Demographics, Plaque Characteristics, and Clinical Outcomes

Although most of the acute symptomatic patients with mild intracranial stenosis had favorable outcomes (*N* = 221, 75.4%), 24.6% (*N* = 72; 63 culprit) had a poor outcome with mRS ≥2 at 90 days after discharge (Table 2). Univariable analysis indicated that hypertensive patients with favorable outcomes had shorter duration of hypertension than those with unfavorable outcomes (5.2 ± 6.2 vs. 12.9 ± 10.7; *P* < 0.05). As with plaque type, significant differences in plaque volume, plaque burden, MLA, remodeling ratio, and enhancement ratio were found between these two groups (all *P* < 0.05). Moreover, patients with nonstrict control of blood pressure, lower plaque enhancement grade, and eccentric lesions had significantly more unfavorable outcomes (*P* < 0.05).

**TABLE 2:** Univariable and Multivariable Analysis on Clinical Outcome After Medical Management

	Univariable Analysis, Mean ± SD or *N* (%)			Multivariable Logistic Regression	
	Favorable (mRS 0–2) *N* = 221	Unfavorable (mRS 3–6) *N* = 72	*P*‐Value	Odds ratio (95% CI)	*P*‐Value
Clinical characteristics					
Age	58.2 ± 10.0	61.9 ± 10.9	**0.012**	0.95 (0.85–1.06)	0.377
Male	149 (67.4)	51 (70.8)	0.589		
Hypertension	97 (43.9)	52 (72.2)	**<0.001**	2.79 (1.47–5.29)	0.051
Hypertension duration	4.5 ± 6.2	13.6 ± 11.1	**<0.001**	1.19 (1.09–1.29)	**<0.001**
Hypertension management					
No control	54 (55.7)	9 (17.3)	**<0.001**	2.49 (1.18–5.26)	**0.017**
Partial control	37 (38.1)	21 (40.4)			
Strict control	6 (6.2)	22 (42.3)			
Diabetes mellitus	72 (32.6)	11 (15.3)	**0.005**	0.57 (0.15–2.14)	0.406
Diabetes duration	5.0 ± 6.3	4.1 ± 8.4	0.297		
Hyperlipidemia	65 (29.4)	15 (20.8)	0.156		
Hyperlipidemia duration	2.4 ± 3.6	2.2 ± 5.1	0.701		
Smoking	92 (41.6)	21 (29.2)	0.059		
Smoking duration	8.2 ± 10.9	10.7 ± 18.3	0.172		
NIHSS	3.2 ± 2.5	3.2 ± 2.4	0.989		
Location, *N* (%)					
MCA	119 (53.8)	50 (69.4)	**0.020**		
BA	102 (46.2)	22 (30.6)			
hrMRI characteristics					
Enhancement ratio (%)	19.8 ± 20.1	13.1 ± 19.2	**0.011**	0.01 (0.001–0.157)	**0.001**
Enhancement grade					
Grade 0, <15%	67 (30.3)	42 (58.3)	**<0.001**	0.28 (0.07–1.17)	0.081
Grade 1, 15%–50%	129 (58.4)	22 (30.6)			
Grade 2, >50%	25 (11.3)	8 (11.1)			
Stenosis (%)	35.1 ± 11.8	33.9 ± 9.9	0.462		
Intraplaque hemorrhage	32 (14.5)	12 (16.7)	0.652		
MLA (mm^2^)	3.9 ± 3.2	3.1 ± 2.5	**0.040**	1.34 (1.00–1.78)	0.067
Plaque volume (mm^3^)	31.9 ± 17.7	27.5 ± 13.3	**0.029**	0.97 (0.94–1.01)	0.056
Plaque burden (%)	75.3 ± 11.7	80.7 ± 8.7	**0.001**	28.7 (13.0–63.2)	0.104
Remodeling ratio (%)	107.1 ± 40.8	119.7 ± 68.2	0.059		
Remodeling type					
Positive remodeling	105 (56.5)	27 (45.0)	0.122		
Negative remodeling	81 (43.5)	33 (55.0)			
Eccentricity	131 (59.3)	65 (90.3)	**<0.001**	3.3 (0.9–11.9)	0.073

The bold numbers represented *P*‐value <0.05.

hrMRI = higher resolution magnetic resonance imaging; MLA = minimal luminal area; mRS = modified Rankin Scale; NIHSS = National Institutes of Health Stroke Scale.

The multivariable logistic regression showed that only hypertension duration (OR 1.24; 95% CI 1.12–1.37; *P* < 0.05), hypertension management (OR 2.53; 95% CI 1.13–5.68; *P* < 0.05), and enhancement ratio (OR 0.015; 95% CI 0.001–0.105; *P* < 0.05) were independent high‐risk factors to predict the clinical outcomes. The AUCs were 0.789 (95% CI 0.700–0.870), 0.751 (95% CI 0.650–0.831), and 0.682 (95% CI 0.575–0.769), respectively (Fig. 4b). When combined, the AUC increased to 0.925 with accuracy, sensitivity, and specificity being 0.859, 0.874, and 0.825, respectively. The diagnostic performance is shown in Table 3.

**TABLE 3:** The Diagnostic Performance of Logistic Regressions

ROC	AUC	DA	Sensitivity	Specificity	PPV	NPV	LR−	LR+
Lesion type								
Mode	0.829	0.839	0.861	0.792	0.897	0.731	4.135	0.175
Clinical outcome								
Mode	0.925	0.859	0.874	0.825	0.922	0.733	4.992	0.153

DA = diagnosis accuracy; LR− = negative likelihood ratio; LR+ = positive likelihood ratio; NPV = negative predictive value; PPV = positive predictive value.

If only patients with culprit lesion (*N* = 233) were considered, it remained nearly the same that hypertension duration and management together with lesion enhancement ratio were key determinants associated with clinical outcome (170 had favorable outcome and 63 unfavorable). The quantitative comparisons between these two groups were provided in Table S1.

### Hypertension, Hypertension Management, and Clinical Outcomes

In the present study, 149 patients suffered from hypertension and culprit lesions were found in 135 of them. Only 9.6% of these patients (*N* = 28) were subject to strict blood pressure control. Patients without any previous control were younger than those with partial or strict control (55.5 ± 7.9 vs. 66.9 ± 10.5 or 64.0 ± 5.3; unit: year; both *P* < 0.05) (Table 4). In contrast, significantly more (*P* < 0.05) patients without hypertension had a favorable outcome (124/144) than patients with hypertension (97/149). For those without any previous control (*N* = 63), 86% (54/63) achieved functional independence (mRS 0–2) after standard blood pressure lowering therapy, while only 64% patients in the group with partial control (37/58) had a favorable clinical outcome. On the contrary, only 21% (6/28) patients with a previous strict blood pressure control had a favorable outcome.

**TABLE 4:** Comparison of the Clinical and Plaque Characteristics in Hypertension Management

	Hypertension Management, Mean ± SD or *N* (%)			
	No Control, *N* = 63	Partial Control, *N* = 58	Strict Control, *N* = 28	*P*‐Value
Age stage (year)	55.5 ± 7.9	66.9 ± 10.5	64.0 ± 5.3	<0.001
Males	53 (84.1)	42 (72.4)	15 (53.6)	0.009
Culprit plaque	62 (98.4)	53 (91.4)	20 (71.4)	<0.001
Unfavorable outcome	9 (14.3)	21 (36.2)	22 (78.6)	<0.001
Enhancement ratio (%)	21.7 ± 17.6	21.4 ± 21.4	12.9 ± 29.9	0.173
No enhancement	16 (25.4)	20 (34.5)	17 (60.7)	0.003
Intraplaque hemorrhage	6 (9.5)	4 (6.9)	6 (21.4)	0.115
Stenosis ratio (%)	37.6 ± 11.9	31.9 ± 8.8	41.1 ± 6.1	<0.001
MLA (mm^2^)	3.1 ± 2.3	3.8 ± 3.3	3.2 ± 2.8	0.275
Plaque volume (mm^3^)	30.9 ± 16.8	31.3 ± 21.8	29.3 ± 12.9	0.890
Plaque burden (%)	76.8 ± 12.3	78.9 ± 9.5	79.3 ± 11.0	0.490
Remodeling ratio (%)	113.6 ± 68.4	106.6 ± 51.8	120.6 ± 52.3	0.580
Positive remodeling	17 (35.4)	22 (43.1)	11 (57.9)	0.242
Eccentricity	12 (40.0)	24 (77.4)	10 (83.3)	0.002

MLA = minimal luminal area.

**FIGURE 5:**
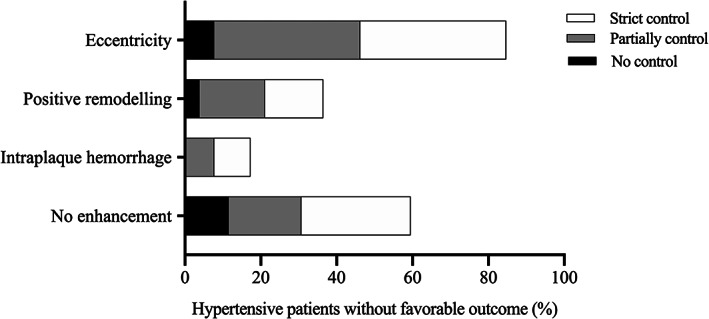
The frequency distribution of the patients with unfavorable outcome on the subgroups of hypertension management with different hrMRI features (no enhancement, IPH, positive remodeling, and eccentricity.

Furthermore, in the 28 patients with a previous strict blood pressure control, 22 had an unfavorable outcome, as shown in Table 4. As shown in Fig. 5, of these 22 patients, lesions in 15 patients did not show any enhancement, five had IPH, eight had positive remodeling, and 20 were eccentric. In those with a partial blood pressure control (*N* = 58), 21 patients achieved a poor outcome. In these 21 patients, 19.0% (*N* = 4) had IPH, 42.9% (*N* = 9) had positive remodeling, 47.6% (*N* = 10) showed no enhancement and 95.2% (*N* = 20) were eccentric. Only nine cases in the subgroup having no hypertensive control achieved an unfavorable outcome. In these, hrMRI showed all plaques were without IPH, six cases were without enhancement, four cases were eccentric, and two showed positive remodeling.

### Reproducibility of hrMRI Analysis

The reliability of hrMRI for the identification of morphological and compositional features in ICAD was assessed through the intra‐ and interobserver agreement analysis. The intra‐ and interobserver agreements for all variables were excellent. The ICCs of the intra‐ and interobserver agreement were shown in Table S2. The ICC of intraobserver agreement was 0.89 ± 0.01 and that of interobserver agreement was 0.91 ± 0.02. The kappa values for IPH identification and eccentricity calculation were 0.92 and 0.90, respectively.

## Discussion

This study revealed a prevalence of about a third (293/967) of intracranial atherosclerotic plaque without substantial stenosis in symptomatic patients (967 is the number of total patients with acute ischemic symptom refers to all of the patients underwent hrMRI with mild to substantial stenosis). Logistic regression analysis indicated that hypertension and plaque enhancement ratio were two independent factors strongly associated with culprit lesions and clinical treatment outcomes; patients without a history of hypertension were found to be very likely to have a nonculprit plaque and a good outcome. For those with uncontrolled or partially controlled hypertension, the standard treatment protocol for risk management was effective. However, the current standard treatment strategy was insufficient for patients with previous strict blood pressure control who had predominantly unfavorable outcomes.

It has been widely reported that hypertension plays an important role in the development of atherosclerosis, which might eventually result in occlusive and artery‐to‐artery embolic strokes.^27^, ^28^, ^29^ Although hypertensive patients with nonstenotic ICAD may have a higher rate of unfavorable outcome compared with those without hypertension, the duration of hypertension and previous management are additional important predictors of outcome when adjusting for all other high‐risk factors, including: age; diabetes mellitus; NIHSS; and hrMRI‐defined plaque features (such as enhancement ratio, enhancement grade, remodeling ratio, and eccentricity). It was found that the longer the duration of the hypertensive status, the higher the incidence of an unfavorable outcome. The possibility of an unfavorable outcome among the individuals with hypertension may be nearly three times than that among nonhypertensive patients (OR = 2.79). The findings remained valid in symptomatic patients with culprit lesions, that is, the hypertension and enhancement ratio were also key determinants for clinic outcome for those with culprit lesions.

This implies that the current standard clinical management for these patient cohorts might be improved by accounting for systematic chronic damage induced by high blood pressure. A longer duration of hypertension might induce more damage to the small cerebral arteries and to nearby brain tissues. It is also possible that unidentified high‐risk factors develop within the period when the blood pressure management is applied and hypertension occurs. Unfavorable outcomes were more likely to occur in a hypertensive cohort with previous strict control than in a cohort without any control. This does not mean that previous strict blood pressure control leads to an unfavorable outcome. On the contrary, our findings may have demonstrated the benefit of previous blood pressure control as it may have postponed symptom occurrence. And it was evidenced by the fact that patients without any control were over 10 years younger than those with. High blood pressure with inadequate previous management was likely the main cause of symptoms for this younger patient cohort, and therefore the standard blood pressure lowering clinical treatment was effective.

In addition to hypertension, hrMRI provided complementary information to assist in differentiating lesion type and in predicting clinical outcomes. Culprit lesions tended to have a higher enhancement ratio, higher prevalence of IPH, smaller MLA, and larger plaque volume than the nonculprit lesions. Unfavorable outcomes were associated with lower enhancement ratio, smaller MLA and plaque volume, higher plaque burden, and larger eccentricity index. Enhancement ratio is a parameter reflecting local inflammation or edema as results of plaque rupture that could be reduced by treatment in the short term.^30^ It is possible that the standard treatment reduced the inflammation effectively in 3 months, while lesion geometric factors such as remodeling ratio and eccentricity were more resistant to the medical management. hrMRI was also useful in predicting clinical outcome in subgroup analysis in patients with hypertension.

According to the trial of ORG 10172 in acute stroke treatment (TOAST) classification,^31^ patients with nonstenotic ICAD and without other identifiable lesions are considered as cryptogenic stroke or embolic stroke of undetermined source (ESUS) rather than large artery atherosclerosis stroke. However, advanced hrMRI might provide the embolic source responsible for the symptoms as positive remodeling might exist in nonstenotic lesions^32^ that are not identifiable by conventional angiography including MRA, CTA, and DSA. Numerous studies^7^, ^8^, ^9^, ^10^, ^11^, ^12^, ^13^, ^14^, ^16^, ^17^, ^18^, ^19^, ^20^, ^21^ have described the association of various plaque characteristics including plaque morphology (such as plaque volume, plaque burden, MLA, remodeling), compositional features (such as IPH) and enhancement with acute stroke in general, regardless of the degree of luminal stenosis. Researchers have reported an urgent need to differentiate nonstenotic atherosclerosis from ESUS for optimal treatment and prevention strategies.^33^ A recent systematic review^8^ showed that over half of ischemic stroke patients without significant stenosis might have a culprit intracranial plaque identifiable on hrMRI. Moreover, the discrepancy between hrMRI and conventional angiography such as MRA was particularly evident in patients with mild stenosis, suggesting that hrMRI is more sensitive for detecting early ICAD.^34^, ^35^ hrMRI allows for the characterization of high‐risk plaques that can be used to identify culprit lesions more accurately,^36^ especially using gadolinium contrast enhancement within the plaque which has been shown to correlate with fibrous cap inflammation and rupture, as confirmed in histological specimens.^17^ Results obtained in this study are in agreement with previous studies in that atherosclerotic plaques in the vessel supplying the infarction territory may show strong enhancement.^14^, ^18^

### Limitations

Prehospitalization information on management of diabetes and hyperlipidemia was not available and analysis on these aspects was therefore not performed. Although these factors might contribute to the differentiation of lesion type and treatment outcome, compared with hypertension, far fewer patients in this study had diabetes and hyperlipidemia. Moreover, there was no significant difference in the distribution of patients with diabetes or hyperlipidemia between the groups with different type lesions or different clinic outcomes. Furthermore, 28 patients had both diabetes and hypertension, while 27 patients had hyperlipidemia and hypertension, though none of the patients had all three characteristics. Glucose and lipid levels in these patients should be managed following the same pattern as the blood pressure. Therefore, the missing information of the prehospitalization management of diabetes and hyperlipidemia should have a limited impact on the conclusions of this study. Further study limitations include: 1) this was a single‐center study limited to Chinese populations; 2) our analysis was performed using two‐dimensional imaging data. It is possible that the thinner slices that could be achieved with 3D imaging could allow better characterization of plaque features by reducing partial volume effects; 3) longer follow‐up should be performed to ensure the effectiveness of the current treatment strategy in patients with unfavorable outcomes in 3 months; and 4) patients might not remember their exact history of blood pressure management and the uncertainty in the classification of blood pressure control might exist.

## Conclusions

Hypertension and plaque enhancement were two independent factors strongly associated with culprit lesions and clinical treatment outcomes. hrMRI provided incremental value over traditional risk factors in identifying higher risk intracranial atherosclerosis with mild luminal stenosis. And current standard clinical management might be sub‐optimal for the patients with previous strict blood pressure control.

## Author Contributions

Drs Zhang Shi and Ming Zhao contributed to the study concept and design, analysis and interpretation of data, drafting/revising the manuscript for content, and statistical analysis. Dr Jing Li was involved in analysis and interpretation of data. Zakaria Meddings contributed to the revision of the language of the manuscript. Dr Yibing Shi performed statistical analysis and interpretation of data. Drs Tao Jiang and Qi Liu took part in the study concept and design, analysis, and interpretation of data. Drs Benqiang Deng, Jianping Lu, and Zhongzhao Teng contributed to the study concept and design, acquisition, analysis, and revising the manuscript for content.

## Supporting information


Table S1.
