# Supplementary material for: Association of Hypertension With Both Occurrence and Outcome of Symptomatic Patients With Mild Intracranial Atherosclerotic Stenosis: A Prospective Higher Resolution Magnetic Resonance Imaging Study
Source: J Magn Reson Imaging. 2021 Mar 10;54(1):76–88. doi: 10.1002/jmri.27516 (PMC8319792; doi:10.1002/jmri.27516)
Supplement: Supplementary file 1 — Table S1. [file JMRI-54-76-s001.docx]

**_Supplementary files_**

**Title:** Association of Hypertension With Both Occurrence and Outcome of Symptomatic Patients With Mild Intracranial Atherosclerotic Stenosis: A Prospective Higher Resolution Magnetic Resonance Imaging Study

**Supplemental Table I. Univariable** **and multivariable analysis on clinical outcome of the patients with culprit lesions after medical management**

|  | Univariable analysis  [Mean ± SD or n (%)] | | |  | Multivariable logistic regression | |
| --- | --- | --- | --- | --- | --- | --- |
|  | Favorable (mRS 0-2)  n=170 | Unfavorable  (mRS 3-6) n=63 | *P* value |  | Odd ratio (95% CI) | *P* value |
| **Clinical characteristics** |  |  |  |  |  |  |
| Age | 57.7±10.6 | 61.8±11.4 | **0.015** |  | 0.97 (0.91-1.03) | 0.279 |
| Male | 113 (66.5) | 47 (74.6) | 0.235 |  |  |  |
| Hypertension | 90 (52.9) | 45 (71.4) | **0.011** |  | 2.79 (1.47-5.29) | 0.051 |
| Hypertension duration | 5.2±6.2 | 12.9±10.7 | **<0.001** |  | 1.24 (1.12-1.37) | **<0.001** |
| Hypertension management |  |  | **<0.001** |  | 2.53 (1.13-5.68) | **0.024** |
| No control | 53 (58.9) | 9 (20.0) |  |  |  |  |
| Partial control | 32 (35.6) | 21 (46.7) |  |  |  |  |
| Strict control | 5 (5.6) | 15 (33.3) |  |  |  |  |
| Diabetes mellitus | 52 (30.6) | 11 (17.5) | **0.045** |  | 0.66 (0.14-3.12) | 0.596 |
| Diabetes duration | 5.0±6.7 | 4.6±8.8 | 0.693 |  |  |  |
| Hyperlipidemia | 39 (22.9) | 11 (17.5) | 0.365 |  |  |  |
| Hyperlipidemia duration | 1.6±3.1 | 2.0±4.9 | 0.416 |  |  |  |
| Smoking | 68 (40.0) | 21 (33.3) | 0.352 |  |  |  |
| Smoking duration | 8.2±10.9 | 11.2±19.1 | 0.121 |  |  |  |
| NIHSS | 3.4±2.7 | 3.3±2.5 | 0.757 |  |  |  |
| Location |  |  | **0.020** |  | 0.78 (0.17-3.57) | 0.750 |
| MCA | 119 (53.8) | 50 (69.4) |  |  |  |  |
| BA | 102 (46.2) | 22 (30.6) |  |  |  |  |
| **hrMRI characteristics** |  |  |  |  |  |  |
| Enhancement ratio (%) | 22.7±21.0 | 14.2±19.1 | **0.005** |  | 0.015 (0.001-0.105) | **0.001** |
| Enhancement grade |  |  | **0.001** |  | 1.04 (0.15-7.03) | 0.969 |
| Grade 0, < 15% | 49 (28.8) | 35 (55.6) |  |  |  |  |
| Grade 1, 15%-50% | 96 (56.5) | 20 (31.7) |  |  |  |  |
| Grade 2, >50% | 25 (14.7) | 8 (12.7) |  |  |  |  |
| Stenosis (%) | 35.6±11.8 | 32.8±9.9 | 0.088 |  |  |  |
| Intraplaque haemorrhage | 32 (18.8) | 11 (17.5) | 0.812 |  |  |  |
| MLA (mm^2^) | 4.2±3.5 | 3.3±2.7 | **0.041** |  | 1.43 (1.01-2.04) | 0.067 |
| Plaque volume (mm^3^) | 33.8±19.3 | 28.3±13.6 | **0.038** |  | 0.97 (0.93-1.01) | 0.056 |
| Plaque burden (%) | 74.9±11.3 | 80.0±8.7 | **<0.001** |  | 29.4 (14.1-67.3) | 0.152 |
| Remodelling ratio (%) | 108.1±45.1 | 125.2±70.8 | **0.030** |  | 0.61 (0.24-1.56) | 0.298 |
| Remodelling type |  |  | 0.773 |  |  |  |
| Positive remodelling | 75 (51.4) | 25 (49.0) |  |  |  |  |
| Negative remodelling | 71 (48.6) | 26 (51.0) |  |  |  |  |
| Eccentricity | 104 (61.2) | 56 (88.9) | **<0.001** |  | 2.54 (0.67-9.75) | 0.173 |

NIHSS= National Institutes of Health Stroke Scale; mRS= modified Rankin scale

MCA=Middle cerebral artery; BA=Basilar artery; MLA = Minimum luminal area

**Supplemental Table-II. The analysis of intra- and inter-observer reproducibility**

|  | Intra-class coefficient (95% CI) | |
| --- | --- | --- |
|  | Intra-observer | Inter-observer |
| Enhancement ratio | 0.901 (0.884-0.915) | 0.918 (0.891-0.934) |
| Stenosis | 0.889 (0.871-0.902) | 0.913 (0.895-0.938) |
| Plaque burden | 0.892 (0.879-0.911) | 0.909 (0.884-0.926) |
| Plaque volume | 0.897 (0.885-0.913) | 0.915 (0.897-0.938) |
| Minimum luminal area | 0.902 (0.887-0.925) | 0.920 (0.898-0.942) |
| Remodelling ratio | 0.896 (0.878-0.910) | 0.908 (0.878-0.935) |
| Eccentricity | 0.904 | 0.921 |
| Intraplaque hemorrhage (Kappa) | 0.916 | 0.923 |
